# Association Between Acidosis Soon After Reperfusion and Contrast‐Induced Nephropathy in Patients With a First‐Time ST‐Segment Elevation Myocardial Infarction

**DOI:** 10.1161/JAHA.117.006380

**Published:** 2017-08-23

**Authors:** Masaomi Gohbara, Azusa Hayakawa, Yusuke Akazawa, Shuta Furihata, Ai Kondo, Yusuke Fukushima, Sakie Tomari, Tsutomu Endo, Kazuo Kimura, Kouichi Tamura

**Affiliations:** ^1^ Division of Cardiology Saiseikai Yokohamashi Nanbu Hospital Yokohama Japan; ^2^ Division of Cardiology Yokohama City University Medical Center Yokohama Japan; ^3^ Department of Medical Science and Cardiorenal Medicine Yokohama City University Graduate School of Medicine Yokohama Japan

**Keywords:** acidosis, contrast‐induced nephropathy, ST‐segment elevation myocardial infarction, Nephrology and Kidney, Myocardial Infarction

## Abstract

**Background:**

Contrast‐induced nephropathy (CIN) is associated with poor outcomes in patients with acute myocardial infarction. However, the predictors of CIN have yet to be fully elucidated.

**Methods and Results:**

The study included 273 consecutive patients with a first‐time ST‐segment elevation myocardial infarction who underwent reperfusion within 12 hours of symptom onset. The exclusion criteria were hemodialysis, mechanical ventilation, or previous coronary artery bypass grafting. All patients underwent arterial blood gas analysis soon after reperfusion. CIN was defined as an increase of 0.5 mg/dL in serum creatinine or a 25% increase from baseline between 48 and 72 hours after contrast medium exposure. Acidosis was defined as an arterial blood pH <7.35. CIN was observed in 35 patients (12.8%). Multivariable logistic regression analysis with forward stepwise algorithm revealed a significant association between CIN and the following: reperfusion time, the prevalence of hypertension, peak creatine kinase‐MB, high‐sensitivity C‐reactive protein on admission, and the incidence of acidosis (*P*<0.05). Multivariable logistic regression analysis revealed that the incidence of acidosis was associated with CIN when adjusted for age, male sex, body mass index, amount of contrast medium used, estimated glomerular filtration rate on admission, glucose level on admission, high‐sensitivity C‐reactive protein on admission, and left ventricular ejection fraction (*P*<0.05). Moreover, the incidence of acidosis was associated with CIN when adjusted for the Mehran CIN risk score (odds ratio: 2.229, *P*=0.049).

**Conclusions:**

The incidence of acidosis soon after reperfusion was associated with CIN in patients with a first‐time ST‐segment elevation myocardial infarction.

**Clinical Perspective**

**What Is New?**

- This is the first study to demonstrate a relationship between the incidence of acidosis and contrast‐induced nephropathy in patients with a first‐time ST‐segment elevation myocardial infarction.

**What Are the Clinical Implications?**

- Since acidosis defined by arterial blood pH is a noninvasive and simple method, it is clinically useful and helpful as a marker of risk stratification for contrast‐induced nephropathy.

## Introduction

Contrast‐induced nephropathy (CIN) is associated with poor outcomes in patients with acute myocardial infarction (AMI).^1^, ^2^, ^3^, ^4^, ^5^, ^6^ CIN is caused by renal vasoconstriction, renal hypoxia, and direct toxicity on tubular epithelial cells caused by contrast medium exposure. Many factors have been reported as predictors of CIN (advanced age, female sex, chronic kidney disease, heart failure, reduced ejection fraction, anemia, high‐sensitivity C‐reactive protein [hs‐CRP], glucose level on admission, diabetes mellitus, high doses of contrast medium, etc).^7^, ^8^, ^9^, ^10^, ^11^, ^12^, ^13^, ^14^ In addition, some risk scoring systems have been used to predict CIN in a clinical setting.^7^, ^15^ However, the predictors of CIN are yet to be fully elucidated.

AMI leading to congestive heart failure or cardiogenic shock may cause renal dysfunction attributable to acute kidney injury (AKI). In patients with AMI, acute kidney injury is caused not only by contrast medium exposure but also by changes in hemodynamic status. Although there are some compounding factors in the definition of CIN in patients with AMI, CIN is generally defined as an increase of 0.5 mg/dL in serum creatinine or a 25% increase from baseline between 48 and 72 hours after contrast medium exposure. In the clinical setting, blood pressure or Killip classification are commonly used to assess the hemodynamic status of patients with AMI. However, it can often be difficult to evaluate hemodynamic status precisely based on such parameters alone. Right heart catheterization is considered the best method for evaluating hemodynamic status precisely; however, it is an invasive method. On the other hand, arterial blood gas (ABG) analysis, which is a noninvasive and simple method, can be used to evaluate acid–base status, directly reflecting hemodynamic status. We hypothesized that the incidence of acidosis evaluated using ABG could predict CIN in patients with AMI.

## Methods

### Patients

**Figure 1:**
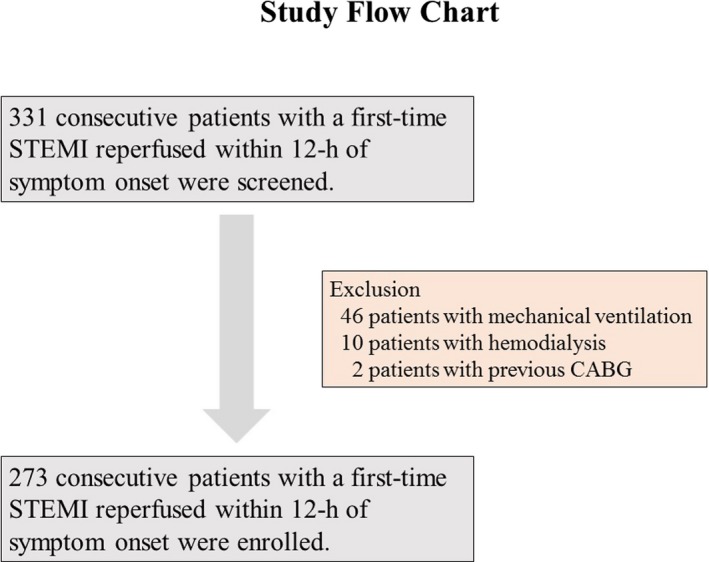
Study flow chart. Of the 331 screened patients, 273 were enrolled in the present study. CABG indicates coronary artery bypass grafting; STEMI, ST‐segment elevation myocardial infarction.

This was a retrospective study conducted between October 2010 and March 2016. A total of 331 consecutive patients, who had undergone emergency coronary angiography and had a significant stenotic culprit lesion, were screened. All patients had a first‐time ST‐segment elevation myocardial infarction (STEMI) and were reperfused within 12 hours of symptom onset in Saiseikai Yokohamashi Nanbu Hospital, Japan. STEMI was defined as chest pain lasting for at least 30 minutes accompanied by new ST‐segment elevation and a rise in cardiac‐specific troponin levels as a value exceeding the 99th percentile of a normal reference population.^16^ ST‐segment elevation was defined as new ST‐segment elevation at the J point in at least 2 contiguous leads of 0.2 mV in men or 0.15 mV in women in leads V2 to V3, or of 0.1 mV in other leads. New left bundle‐branch block was considered equivalent to STEMI. We excluded patients with any of the following characteristics: mechanical ventilation, hemodialysis, or a history of previous coronary‐artery bypass grafting. A total of 273 patients met the eligibility criteria and were enrolled in the study. Forty‐six patients with mechanical ventilation, 10 patients undergoing hemodialysis, and 2 patients with previous coronary‐artery bypass grafting were excluded (Figure 1). Of the 273 patients, 243 underwent stent implantation, 8 underwent balloon angioplasty only, 17 underwent thrombus aspiration only, 2 with spontaneous reperfusion underwent coronary‐artery bypass grafting, and 3 with spontaneous reperfusion received medical therapy only. We divided the patients into 2 groups according to the incidence of CIN (ie, those who developed CIN and those who did not). Following admission, 5000 U of unfractionated heparin was administered to patients. Subsequently, they received aspirin (200 mg loading dose, followed by 100 mg/day), and either clopidogrel (300 mg loading dose, followed by 75 mg/day) or prasugrel (20 mg loading dose, followed by 3.75 mg/day as prasugrel is in general use in Japan). Glycoprotein (GP) IIb/IIIa inhibitors were not used as they have not been approved for use in Japan. The study protocol was approved by the Saiseikai Yokohamashi Nanbu Hospital Institutional Review Board and the subjects gave informed consent.

### Blood Sampling

Biochemistry data including creatine phosphokinase and creatine kinase MB were evaluated on admission, at 3‐hour intervals during the first 24 hours, and then daily during the first 3 days following admission. Glucose levels, hemoglobin A1c, and hs‐CRP were evaluated on admission. Serum creatinine and estimated glomerular filtration rate (eGFR) were evaluated daily during the first 3 days according to the following formula.^17^

$$\text{eGFR}\;\left(\text{mL/min per}\;1.73\;m^{2}\right)=194\times \text{Serum}\;{\text{creatinine}}^{-1.094}\times {\text{Age}}^{-0.287}\times 0.739\;\left(\text{if female}\right)$$

CIN was defined as an increase of 0.5 mg/dL in serum creatinine or a 25% increase from baseline between 48 and 72 hours after contrast medium exposure.

### ABG Protocol

Shortly after reperfusion, blood samples were obtained from the sheath. All patients underwent ABG analysis. Arterial blood pH, partial pressure of arterial oxygen, partial pressure of arterial carbon dioxide, hydrogen carbonate ion (HCO^3−^), and base excess were evaluated. Acidosis was defined as an arterial blood pH <7.35. Metabolic acidosis was defined as an arterial blood pH <7.35 with HCO^3−^ <22 mmol/L. Respiratory acidosis was defined as an arterial blood pH <7.35 with partial pressure of arterial carbon dioxide level >45 mm Hg.

### Two‐Dimensional Echocardiography Protocol

All patients underwent 2‐dimensional echocardiography on admission, immediately after reperfusion. All images were obtained with a commercial ultrasound system in the parasternal (long‐ and short‐axis) and 3‐apical (2‐, 3‐, and 4‐chamber) views with a 3‐MHz phased array probe. Grayscale images were obtained at a frame rate of ≥50 per second. The images were independently interpreted by experienced sonographers blinded to the angiographic and clinical data. Complete 2‐dimensional, color, pulsed, and continuous‐wave Doppler echocardiography was obtained according to standard techniques. Left ventricular ejection fraction (LVEF) and transmitral deceleration time were obtained.

### Risk Scoring of CIN

Many factors have been reported as predictors of CIN. Recently, Mehran et al demonstrated a simple risk score (Mehran risk score) for CIN after percutaneous coronary intervention.^7^ It was calculated by evaluating the presence of hypotension, congestive heart failure, anemia, and diabetes mellitus, the use of intra‐aortic balloon pump, age >75 years, the amount of contrast medium, and the basal renal function. We calculated Mehran risk scores and compared them with acidosis to predict CIN in the present study.

We investigated whether the incidence of acidosis soon after reperfusion was a predictor of CIN when adjusted for well‐known predictors or risk scoring system. Additionally, we investigated the association between the incidence of CIN and in‐hospital mortality rate.

### Statistical Analysis

Continuous variables were expressed as means±SD for normally distributed variables, and as medians (25th to 75th percentiles) for variables with skewed distributions. Differences between the groups were tested using Student *t* test for normally distributed variables, the Mann–Whitney test for variables with skewed distributions, and the χ^2^ test or Fisher exact test as appropriate for categorical variables. Univariate variables to predict CIN were analyzed, and variables with *P*<0.2 on univariate analysis were entered into a multivariable logistic analysis with forward stepwise algorithm. Peak creatine phosphokinase, serum creatinine, metabolic acidosis, and respiratory acidosis were excluded because of the effect of confounding factors. We then established multivariable logistic regression analysis using forced inclusion models for the prediction of CIN (model‐1: age, male sex, body mass index, and the incidence of acidosis; model‐2: amount of contrast medium, eGFR on admission, and the incidence of acidosis; model‐3: glucose level on admission, hs‐CRP on admission, the incidence of acidosis, and LVEF; model‐4: the incidence of acidosis and Mehran risk score). All statistical tests were 2‐tailed, and *P*<0.05 was considered to indicate statistical significance. SPSS version 18.0 (SPSS Japan Inc, Tokyo, Japan) was used for all statistical analyses.

## Results

### Baseline and Clinical Characteristics

Baseline and clinical characteristics are shown in Table 1. CIN was observed in 35 patients (12.8%). Patients with CIN had higher age, longer reperfusion times, higher prevalence of hypertension, higher glucose level on admission, higher peak creatine phosphokinase and creatine kinase MB, and higher hs‐CRP on admission than those without CIN (all *P*<0.05). In addition, patients with CIN had higher incidence of acidosis (especially respiratory acidosis) than those without CIN (*P*=0.014). Two‐dimensional echocardiography findings revealed that patients with CIN had slightly lower LVEF than those without CIN (55% versus 57%, *P*=0.049). Moreover, patients with CIN had higher Mehran risk score of CIN than those without CIN (6 versus 4, *P*=0.022). Conversely, although in previous studies, the prevalence of Killip class >1 tended to be associated with CIN, it did not reach statistical significance in the present study (*P*=0.050). Furthermore, in the present study, there were no significant differences between the 2 groups regarding the prevalence of diabetes mellitus, amount of contrast medium, serum creatinine, and eGFR on admission, which were the well‐known predictors of CIN previously (*P*=0.728, 0.970, 0.305, and 0.451, respectively).

**Table 1:** Baseline and Clinical Characteristics

Variables	With CIN (n=35)	Without CIN (n=238)	*P* Value
Age, y	75 (64–80)	68 (60–76)	0.041
Male, n (%)	27 (77)	188 (79)	0.803
Body mass index, kg/m^2^	23 (22–25)	24 (22–26)	0.708
Killip class >1, n (%)	8 (23)	25 (11)	0.050
Reperfusion time, h	3.9 (2.4–6.0)	2.8 (2.0–4.0)	0.004
Culprit artery			0.422
Left anterior descending artery, n (%)	20 (57)	113 (47)	
Right coronary artery, n (%)	9 (26)	88 (37)	
Left circumflex artery, n (%)	6 (17)	37 (16)	
Number of diseased vessels, n (%)			0.411
1	15 (43)	123 (51)	
2	15 (43)	75 (32)	
3	5 (14)	40 (17)	
Amount of contrast medium, mL	133 (100–200)	135 (104–160)	0.970
Current smoker, n (%)	15 (43)	88 (37)	0.503
Hypertension, n (%)	27 (77)	128 (54)	0.009
Dyslipidemia, n (%)	25 (71)	195 (82)	0.142
Diabetes mellitus, n (%)	11 (31)	68 (29)	0.728
Glucose level on admission, mg/dL	192 (153–213)	152 (125–201)	0.004
Hemoglobin A1c, %	5.8 (5.7–6.6)	6.0 (5.7–6.5)	0.614
Peak CPK, IU/L	2323 (1298–6142)	1535 (695–3112)	0.012
Peak CK‐MB, IU/L	233 (184–599)	172 (74–334)	0.008
hs‐CRP on admission, mg/dL	0.20 (0.07–0.94)	0.10 (0.05–0.25)	0.008
Serum creatinine, mg/dL	0.82 (0.64–1.27)	0.90 (0.80–1.05)	0.305
eGFR on admission, mL/min per 1.73 m^2^	68 (43–84)	63 (52–71)	0.451
Acidosis, n (%)	12 (34)	40 (17)	0.014
Metabolic acidosis, n (%)	7 (20)	20 (8)	0.061
Respiratory acidosis, n (%)	5 (14)	8 (3)	0.016
LVEF, %	55 (47–60)	57 (51–62)	0.049
DcT, ms	191 (169–217)	209 (172–259)	0.144
Mehran risk score	6 (4–11)	4 (1–8)	0.022

Data are displayed as mean±SD or number (percentage) or median (range). CIN indicates contrast‐induced nephropathy; CK‐MB, creatine kinase MB; CPK, creatine phosphokinase; DcT, deceleration time; eGFR, estimated glomerular filtration rate; hs‐CRP, high‐sensitivity C‐reactive protein; LVEF, left ventricular ejection fraction.

### In‐Hospital Mortality

**Figure 2:**
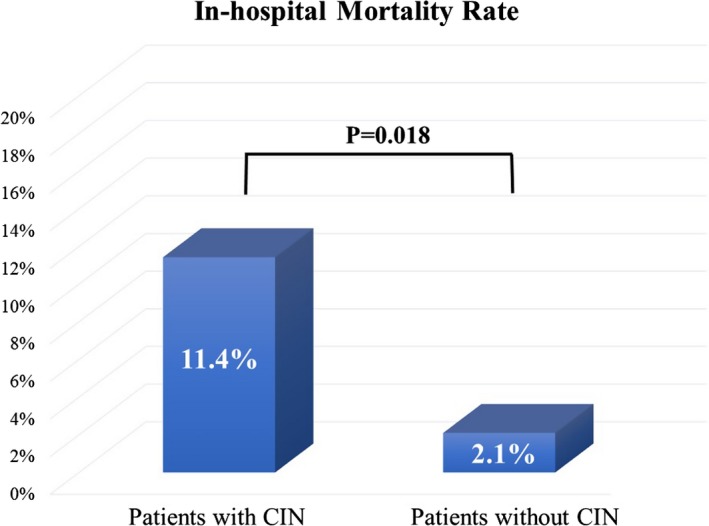
The association between the incidence of CIN and in‐hospital mortality rate. In‐hospital mortality rate was higher in patients with CIN (11.4%, 4 of 35 patients) than that in patients without CIN (2.1%, 5 of 238 patients) (*P*=0.018). CIN indicates contrast‐induced nephropathy.

In‐hospital mortality rate was higher in patients with CIN (11.4%, 4 of 35 patients) than in patients without CIN (2.1%, 5 of 238 patients) (*P*=0.018, Figure 2).

### Univariate Logistic Regression Analysis to Predict CIN

Univariate logistic analysis revealed that there was a significant association between CIN and the following: Killip class >1, reperfusion time, the prevalence of hypertension, glucose level on admission, peak creatine kinase MB, hs‐CRP on admission, the incidence of acidosis, LVEF, and Mehran risk score (all *P*<0.05, Table 2). In contrast, age, male sex, body mass index, and dyslipidemia were not associated with CIN in the present study (*P*=0.051, 0.803, 0.648, and 0.147, respectively, Table 2). In addition, the amount of contrast medium and eGFR on admission, which were the well‐known predictors of CIN previously, were not associated with CIN in the present study (*P*=0.740 and 0.592, Table 2).

**Table 2:** Univariate and Multivariable Logistic Regression Analyses to Predict CIN

Variables	Univariate			Multivariable (Stepwise)			Multivariable (Forced Inclusion Model 1)		
	OR	95% CI	*P* Value	OR	95% CI	*P* Value	OR	95% CI	*P* Value
Age, per 1 y	1.030	1.000 to 1.062	0.051			0.227	1.034	1.000 to 1.069	0.053
Male	0.898	0.384 to 2.097	0.803	Not selected			1.299	0.522 to 3.229	0.574
Body mass index, per 1 kg/m^2^	0.975	0.875 to 1.087	0.648	Not selected			1.016	0.902 to 1.145	0.618
Killip class >1	2.524	1.035 to 6.154	0.042			0.664	···		
Reperfusion time, per 1 h	1.217	1.077 to 1.375	0.002	1.202	1.044 to 1.385	0.011	···		
Amount of contrast medium, per 1 mL	1.001	0.994 to 1.008	0.740	Not selected			···		
Hypertension	2.900	1.266 to 6.645	0.012	3.168	1.292 to 7.772	0.012	···		
Dyslipidemia	0.551	0.247 to 1.232	0.147			0.337	···		
Glucose level on admission, per 1 mg/dL	1.006	1.001 to 1.011	0.017			0.093	···		
Peak CK‐MB, per 1 IU/L	1.002	1.001 to 1.004	0.001	1.002	1.000 to 1.004	0.017	···		
hs‐CRP on admission, per 1 mg/dL	1.246	1.025 to 1.514	0.027	1.245	1.034 to 1.498	0.020	···		
eGFR on admission, per 1 mL/min per 1.73 m^2^	1.005	0.986 to 1.026	0.592	Not selected			···		
Acidosis	2.583	1.188 to 5.613	0.017	2.713	1.113 to 6.616	0.028	2.548	1.160 to 5.593	0.020
LVEF, %	0.959	0.921 to 0.997	0.035			0.712	···		
Mehran risk score	1.081	1.016 to 1.150	0.014			0.458	···		

Variables	Multivariable (Forced Inclusion Model 2)			Multivariable (Forced Inclusion Model 3)			Multivariable (Forced Inclusion Model 4)		
	OR	95% CI	*P* Value	OR	95% CI	*P* Value	OR	95% CI	*P* Value
Age, per 1 y	···			···			···		
Male	···			···			···		
Body mass index, per 1 kg/m^2^	···			···			···		
Killip class >1	···			···			···		
Reperfusion time, per 1 h	···			···			···		
Amount of contrast medium, per 1 mL	1.001	0.994 to 1.008	0.871	···			···		
Hypertension	···			···			···		
Dyslipidemia	···			···			···		
Glucose level on admission, per 1 mg/dL	···			1.005	1.000 to 1.010	0.064	···		
Peak CK‐MB, per 1 IU/L	···			···			···		
hs‐CRP on admission, per 1 mg/dL	···			1.204	1.007 to 1.440	0.042	···		
eGFR on admission, per 1 mL/min per 1.73 m^2^	1.009	0.989 to 1.030	0.394	···			···		
Acidosis	2.721	1.235 to 5.995	0.013	2.452	1.095 to 5.495	0.029	2.229	1.004 to 4.949	0.049
LVEF, %	···			0.971	0.932 to 1.012	0.167	···		
Mehran risk score	···			···			1.069	1.003 to 1.139	0.041

The table lists all the analyzed variables. Forced inclusion model 1: age, male, body mass index, and the incidence of acidosis. Forced inclusion model 2: amount of contrast medium, eGFR on admission, and the incidence of acidosis. Forced inclusion model 3: glucose level on admission, hs‐CRP on admission, the incidence of acidosis, and LVEF. Forced inclusion model 4: the incidence of acidosis and Mehran risk score. CI indicates confidence interval; CIN, contrast‐induced nephropathy; CK‐MB, creatine kinase MB; eGFR, estimated glomerular filtration rate; hs‐CRP, high‐sensitivity C‐reactive protein; LVEF, left ventricular ejection fraction; OR, odds ratio.

### Multivariable Logistic Regression Analysis to Predict CIN

A multivariable logistic analysis with forward stepwise algorithm and *P*<0.2 on univariate analysis revealed that reperfusion time, the prevalence of hypertension, peak creatine kinase MB, hs‐CRP on admission, and the incidence of acidosis were significantly associated with CIN (all *P*<0.05, Table 2). Multivariable logistic regression analysis by forced inclusion models for prediction of CIN revealed that the incidence of acidosis was associated with CIN when adjusted for age, male sex, body mass index, amount of contrast medium, eGFR on admission, glucose levels on admission, hs‐CRP on admission, and LVEF (model 1 to model 3 in Table 2). In addition, the incidence of acidosis was associated with CIN when adjusted for Mehran risk score (odds ratio: 2.229, 95% CI [confidence interval]: 1.004–4.94, *P*=0.049, model 4 in Table 2). According to these results, the incidence of acidosis was associated with CIN when adjusted for well‐known predictors or risk scoring systems in the present study.

## Discussion

The principal finding of the present study was that the incidence of acidosis soon after reperfusion was associated with CIN when adjusted for other risk factors or risk scoring system in patients with a first‐time STEMI and reperfused within 12 hours of symptom onset. To the best of our knowledge, this is the first study to demonstrate a relationship between the incidence of acidosis and CIN.

### Acidosis

Decreased cardiac output because of AMI may lead to a disturbance in the metabolism of peripheral tissue, resulting in metabolic acidosis. In addition, respiratory failure caused by congestive heart failure resulting from AMI may lead to respiratory acidosis. Acidosis inhibits the myofibrillar responsiveness to Ca^2+^ by decreasing the sensitivity of contractile proteins to Ca^2+^ and by decreasing maximum force.^18^ Acidosis decreases the myocardial contractility and energy production. In addition, the mechanical function is depressed in ischemia not only in anoxic regions of the heart but also in adjacent aerobic regions because of the pH change.^19^ Thus, patients with acidosis are caught in a vicious circle. Some previous reports have demonstrated that the evaluation of acid–base status was helpful in risk stratification in patients with AMI.^20^, ^21^ In addition, Nagai et al demonstrated that acidosis and subsequent inflammation following reperfusion after STEMI played an important role in the development of sustained ventricular tachycardia and ventricular fibrillation.^22^ Moreover, Park et al demonstrated that acidosis was observed in every fifth patient and was a significant predictor of mortality in patients with acute heart failure.^23^ Hence, it is important to evaluate the acid–base status for risk stratification in patients with AMI.

Recently, the association between acidosis and reperfusion injury has been focused upon. Reperfusion injury (ie, myocardial injury resulting in additional myocardial necrosis or arrhythmias) is caused by the restoration of coronary blood flow after an ischemic episode. Several studies have been undertaken in an attempt to overcome this particular issue. Preckel et al demonstrated that reperfusion injury after coronary artery occlusion could be reduced by prolonged local extracellular acidosis in vivo.^24^ In addition, Kitakaze et al demonstrated that temporary acidosis during reperfusion limited the infarct size in dogs.^25^ Moreover, Zhao et al demonstrated that repetitive cycles of briefly interrupted reperfusion at the onset of coronary reflow significantly reduced the infarct size, and they referred to this procedure as postconditioning.^26^ Prolongation of intracellular acidosis during initial reperfusion protects against ischemia, reperfusion–induced hypercontracture, and cell death.^27^ In postconditioning, prolonged temporary acidosis in the first minutes of reperfusion occurs via the activation of Akt and extracellular signal–regulated kinase, which prevents mitochondrial permeability transition pore formation after pH normalization.^28^, ^29^ Furthermore, acidosis and subsequent activation of Na^+^/H^+^ exchange has been shown to modulate the production of endogenous NO in canine ischemic myocardium.^30^ In addition, acidosis leading to increased H^+^ activity may contribute to local regulation of coronary blood flow by altering the vasoactivity of adenosine.^31^ Moreover, the opening of ATP‐sensitive potassium channels in vascular smooth muscle mediates coronary arteriolar dilation during acidosis.^32^ Although these compensatory responses after acidosis contribute to the effects of postconditioning, the significance of acidosis as a marker of risk stratification as shown in the present study differs from that of artificial acidosis as postconditioning.

### Contrast‐induced Nephropathy

Although there are some compounding factors in the definition of CIN in patients with AMI as mentioned above, CIN is a powerful predictor of major adverse clinical events in these patients.^1^, ^2^, ^3^, ^4^, ^5^, ^6^ In the present study, we demonstrated that the in‐hospital mortality rate was higher in patients with CIN than in patients without CIN, and the incidence of acidosis soon after reperfusion was associated with CIN when adjusted for well‐known predictors or risk scoring system. Since ABG analysis is a noninvasive and simple method, it is clinically useful and helpful.

Although acidosis was not only considered a marker of the risk stratification for CIN but also a mediator of CIN because of decreasing myocardial contractility, it is still not clear whether the intervention to prevent acidosis can improve the prognosis. de Brito‐Ashurst et al demonstrated that oral sodium bicarbonate slowed the progression of chronic kidney disease.^33^ Additionally, some previous reports revealed that hydration with sodium bicarbonate improved acidosis as the rapid increase of intravascular volume was more effective than hydration with sodium chloride for prophylaxis of CIN.^34^, ^35^ In contrast, previous reports have shown that hydration with sodium bicarbonate had no clinical superiority in reducing the risk of CIN than that with sodium chloride.^36^, ^37^, ^38^, ^39^ Since there is no evidence that hydration with sodium bicarbonate decreases the risk of hemodialysis or death, the effect of intervention for acidosis prevention may have a limited impact on CIN. Hence, now we conclude that acidosis is useful as a marker of risk stratification for CIN. However, there is a possibility that the use of sodium bicarbonate may decrease the risk of CIN via a rapid increase of intravascular volume in patients with STEMI. If the intervention to acidosis itself has a preferable effect in those patients, we can conclude that the intervention to acidosis itself may be a good option in patients with AMI as well as postconditioning as artificial acidosis. On the other hand, if the intervention to acidosis itself does not have a preferable effect in those patients, we can conclude that acidosis is just a marker of risk stratification. Further studies are needed to draw this conclusion.

### Limitations

Our study had several limitations. First, it was a retrospective observational study, and the study group comprised a relatively small number of patients enrolled at a single center. Second, there was some bias regarding the amount of contrast medium used during reperfusion therapy, as we were aware of the baseline eGFR on admission. Hence, the amount of contrast medium and eGFR on admission might not be predictors of CIN in the present study. In addition, low power might be a possible explanation that some well‐known risk factors including the amount of contrast medium and eGFR on admission failed to reach statistical significance in the present study. Since the amount of contrast medium and eGFR on admission are established predictors of CIN, there is an ethical issue if we conduct a blind study regarding the baseline eGFR. Therefore, it is difficult to completely avoid this problem. However, we thought that our results had significance in clinical practice in which we were aware of the baseline eGFR. Third, since we excluded high‐risk patients such as those who needed mechanical ventilation, hemodialysis, or those who had a history of coronary‐artery bypass grafting, our findings may not apply to these subgroups of patients. However, we believe that our findings are of clinical significance in most patients.

## Conclusion

The incidence of acidosis soon after reperfusion was associated with CIN when adjusted for other risk factors or risk scoring system in patients with a first‐time STEMI who were reperfused within 12 hours of symptom onset.

## Disclosures

None.

## References

[1] Nakahashi H , Kosuge M , Sakamaki K , Kiyokuni M , Ebina T , Hibi K , Tsukahara K , Iwahashi N , Kuji S , Oba MS , Umemura S , Kimura K . Combined impact of chronic kidney disease and contrast‐induced nephropathy on long‐term outcomes in patients with ST‐segment elevation acute myocardial infarction who undergo primary percutaneous coronary intervention. Heart Vessels. 2017;32:22–29. 27106917 10.1007/s00380-016-0836-8

[2] Giacoppo D , Madhavan MV , Baber U , Warren J , Bansilal S , Witzenbichler B , Dangas GD , Kirtane AJ , Xu K , Kornowski R , Brener SJ , Genereux P , Stone GW , Mehran R . Impact of contrast‐induced acute kidney injury after percutaneous coronary intervention on short‐ and long‐term outcomes: pooled analysis from the HORIZONS‐AMI and ACUITY Trials. Circ Cardiovasc Interv. 2015;8:e002475. 26198286 10.1161/CIRCINTERVENTIONS.114.002475

[3] Watabe H , Sato A , Hoshi T , Takeyasu N , Abe D , Akiyama D , Kakefuda Y , Nishina H , Noguchi Y , Aonuma K . Association of contrast‐induced acute kidney injury with long‐term cardiovascular events in acute coronary syndrome patients with chronic kidney disease undergoing emergent percutaneous coronary intervention. Int J Cardiol. 2014;174:57–63. 24726211 10.1016/j.ijcard.2014.03.146

[4] Narula A , Mehran R , Weisz G , Dangas GD , Yu J , Genereux P , Nikolsky E , Brener SJ , Witzenbichler B , Guagliumi G , Clark AE , Fahy M , Xu K , Brodie BR , Stone GW . Contrast‐induced acute kidney injury after primary percutaneous coronary intervention: results from the HORIZONS‐AMI substudy. Eur Heart J. 2014;35:1533–1540. 24603308 10.1093/eurheartj/ehu063

[5] Pyxaras SA , Sinagra G , Mangiacapra F , Perkan A , Di Serafino L , Vitrella G , Rakar S , De Vroey F , Santangelo S , Salvi A , Toth G , Bartunek J , De Bruyne B , Wijns W , Barbato E . Contrast‐induced nephropathy in patients undergoing primary percutaneous coronary intervention without acute left ventricular ejection fraction impairment. Am J Cardiol. 2013;111:684–688. 23261003 10.1016/j.amjcard.2012.11.018

[6] Marenzi G , Lauri G , Assanelli E , Campodonico J , De Metrio M , Marana I , Grazi M , Veglia F , Bartorelli AL . Contrast‐induced nephropathy in patients undergoing primary angioplasty for acute myocardial infarction. J Am Coll Cardiol. 2004;44:1780–1785. 15519007 10.1016/j.jacc.2004.07.043

[7] Mehran R , Aymong ED , Nikolsky E , Lasic Z , Iakovou I , Fahy M , Mintz GS , Lansky AJ , Moses JW , Stone GW , Leon MB , Dangas G . A simple risk score for prediction of contrast‐induced nephropathy after percutaneous coronary intervention: development and initial validation. J Am Coll Cardiol. 2004;44:1393–1399. 15464318 10.1016/j.jacc.2004.06.068

[8] Ando G , Morabito G , de Gregorio C , Trio O , Saporito F , Oreto G . Age, glomerular filtration rate, ejection fraction, and the AGEF score predict contrast‐induced nephropathy in patients with acute myocardial infarction undergoing primary percutaneous coronary intervention. Catheter Cardiovasc Interv. 2013;82:878–885. 23703775 10.1002/ccd.25023

[9] Abe D , Sato A , Hoshi T , Kakefuda Y , Watabe H , Ojima E , Hiraya D , Harunari T , Takeyasu N , Aonuma K . Clinical predictors of contrast‐induced acute kidney injury in patients undergoing emergency versus elective percutaneous coronary intervention. Circ J. 2014;78:85–91. 24107362 10.1253/circj.cj-13-0574

[10] Ando G , de Gregorio C , Morabito G , Trio O , Saporito F , Oreto G . Renal function‐adjusted contrast volume redefines the baseline estimation of contrast‐induced acute kidney injury risk in patients undergoing primary percutaneous coronary intervention. Circ Cardiovasc Interv. 2014;7:465–472. 25027519 10.1161/CIRCINTERVENTIONS.114.001545

[11] Moriyama N , Ishihara M , Noguchi T , Nakanishi M , Arakawa T , Asaumi Y , Kumasaka L , Kanaya T , Miyagi T , Nagai T , Yamane T , Fujino M , Honda S , Fujiwara R , Anzai T , Kusano K , Goto Y , Yasuda S , Ogawa H . Admission hyperglycemia is an independent predictor of acute kidney injury in patients with acute myocardial infarction. Circ J. 2014;78:1475–1480. 24694768 10.1253/circj.cj-14-0117

[12] Lazaros G , Zografos T , Oikonomou E , Siasos G , Georgiopoulos G , Vavuranakis M , Antonopoulos A , Kalogeras K , Tsalamandris S , Tousoulis D . Usefulness of C‐reactive protein as a predictor of contrast‐induced nephropathy after percutaneous coronary interventions in patients with acute myocardial infarction and presentation of a new risk score (Athens CIN Score). Am J Cardiol. 2016;118:1329–1333. 27745963 10.1016/j.amjcard.2016.07.069

[13] Ebisawa S , Kurita T , Tanaka N , Nasu K , Kimura M , Ito T , Kinoshita Y , Tsuchikane E , Terashima M , Suzuki T . Impact of minimum contrast media volumes during elective percutaneous coronary intervention for prevention of contrast‐induced nephropathy in patients with stable coronary artery disease. Cardiovasc Interv Ther. 2016;31:13–20. 26001976 10.1007/s12928-015-0337-1

[14] Lucreziotti S , Centola M , Salerno‐Uriarte D , Ponticelli G , Battezzati PM , Castini D , Sponzilli C , Lombardi F . Female gender and contrast‐induced nephropathy in primary percutaneous intervention for ST‐segment elevation myocardial infarction. Int J Cardiol. 2014;174:37–42. 24698233 10.1016/j.ijcard.2014.03.087

[15] Brown JR , DeVries JT , Piper WD , Robb JF , Hearne MJ , Ver Lee PM , Kellet MA , Watkins MW , Ryan TJ , Silver MT , Ross CS , MacKenzie TA , O'Connor GT , Malenka DJ . Serious renal dysfunction after percutaneous coronary interventions can be predicted. Am Heart J. 2008;155:260–266. 18215595 10.1016/j.ahj.2007.10.007

[16] Thygesen K , Alpert JS , Jaffe AS , Simoons ML , Chaitman BR , White HD , Katus HA , Lindahl B , Morrow DA , Clemmensen PM , Johanson P , Hod H , Underwood R , Bax JJ , Bonow RO , Pinto F , Gibbons RJ , Fox KA , Atar D , Newby LK , Galvani M , Hamm CW , Uretsky BF , Steg PG , Wijns W , Bassand JP , Menasche P , Ravkilde J , Ohman EM , Antman EM , Wallentin LC , Armstrong PW , Simoons ML , Januzzi JL , Nieminen MS , Gheorghiade M , Filippatos G , Luepker RV , Fortmann SP , Rosamond WD , Levy D , Wood D , Smith SC , Hu D , Lopez‐Sendon JL , Robertson RM , Weaver D , Tendera M , Bove AA , Parkhomenko AN , Vasilieva EJ , Mendis S . Third universal definition of myocardial infarction. Circulation. 2012;126:2020–2035. 22923432 10.1161/CIR.0b013e31826e1058

[17] Matsuo S , Imai E , Horio M , Yasuda Y , Tomita K , Nitta K , Yamagata K , Tomino Y , Yokoyama H , Hishida A . Revised equations for estimated GFR from serum creatinine in Japan. Am J Kidney Dis. 2009;53:982–992. 19339088 10.1053/j.ajkd.2008.12.034

[18] Orchard CH , Kentish JC . Effects of changes of pH on the contractile function of cardiac muscle. Am J Physiol. 1990;258:C967–C981. 2193525 10.1152/ajpcell.1990.258.6.C967

[19] Steenbergen C , Deleeuw G , Rich T , Williamson JR . Effects of acidosis and ischemia on contractility and intracellular pH of rat heart. Circ Res. 1977;41:849–858. 21759 10.1161/01.res.41.6.849

[20] Lazzeri C , Valente S , Chiostri M , Picariello C , Gensini GF . Acid‐base imbalance in uncomplicated ST‐elevation myocardial infarction: the clinical role of tissue acidosis. Intern Emerg Med. 2010;5:61–66. 19998062 10.1007/s11739-009-0338-0

[21] Sahu A , Cooper HA , Panza JA . The initial anion gap is a predictor of mortality in acute myocardial infarction. Coron Artery Dis. 2006;17:409–412. 16845247 10.1097/00019501-200608000-00002

[22] Nagai T , Anzai T , Kaneko H , Anzai A , Mano Y , Nagatomo Y , Kohsaka S , Maekawa Y , Kawamura A , Yoshikawa T , Ogawa S . Impact of systemic acidosis on the development of malignant ventricular arrhythmias after reperfusion therapy for ST‐elevation myocardial infarction. Circ J. 2010;74:1808–1814. 20606327 10.1253/circj.cj-10-0229

[23] Park JJ , Choi DJ , Yoon CH , Oh IY , Lee JH , Ahn S , Yoo BS , Kang SM , Kim JJ , Baek SH , Cho MC , Jeon ES , Chae SC , Ryu KH , Oh BH . The prognostic value of arterial blood gas analysis in high‐risk acute heart failure patients: an analysis of the Korean Heart Failure (KORHF) registry. Eur J Heart Fail. 2015;17:601–611. 26096207 10.1002/ejhf.276

[24] Preckel B , Schlack W , Obal D , Barthel H , Ebel D , Grunert S , Thamer V . Effect of acidotic blood reperfusion on reperfusion injury after coronary artery occlusion in the dog heart. J Cardiovasc Pharmacol. 1998;31:179–186. 9475258 10.1097/00005344-199802000-00002

[25] Kitakaze M , Takashima S , Funaya H , Minamino T , Node K , Shinozaki Y , Mori H , Hori M . Temporary acidosis during reperfusion limits myocardial infarct size in dogs. Am J Physiol. 1997;272:H2071–H2078. 9176271 10.1152/ajpheart.1997.272.5.H2071

[26] Zhao ZQ , Corvera JS , Halkos ME , Kerendi F , Wang NP , Guyton RA , Vinten‐Johansen J . Inhibition of myocardial injury by ischemic postconditioning during reperfusion: comparison with ischemic preconditioning. Am J Physiol Heart Circ Physiol. 2003;285:H579–H588. 12860564 10.1152/ajpheart.01064.2002

[27] Inserte J , Barba I , Hernando V , Abellan A , Ruiz‐Meana M , Rodriguez‐Sinovas A , Garcia‐Dorado D . Effect of acidic reperfusion on prolongation of intracellular acidosis and myocardial salvage. Cardiovasc Res. 2008;77:782–790. 18056767 10.1093/cvr/cvm082

[28] Fujita M , Asanuma H , Hirata A , Wakeno M , Takahama H , Sasaki H , Kim J , Takashima S , Tsukamoto O , Minamino T , Shinozaki Y , Tomoike H , Hori M , Kitakaze M . Prolonged transient acidosis during early reperfusion contributes to the cardioprotective effects of postconditioning. Am J Physiol Heart Circ Physiol. 2007;292:H2004–H2008. 17208997 10.1152/ajpheart.01051.2006

[29] Cohen MV , Yang XM , Downey JM . The pH hypothesis of postconditioning: staccato reperfusion reintroduces oxygen and perpetuates myocardial acidosis. Circulation. 2007;115:1895–1903. 17389262 10.1161/CIRCULATIONAHA.106.675710

[30] Kitakaze M , Node K , Takashima S , Asanuma H , Asakura M , Sanada S , Shinozaki Y , Mori H , Sato H , Kuzuya T , Hori M . Role of cellular acidosis in production of nitric oxide in canine ischemic myocardium. J Mol Cell Cardiol. 2001;33:1727–1737. 11549351 10.1006/jmcc.2001.1435

[31] Merrill GF , Haddy FJ , Dabney JM . Adenosine, theophylline, and perfusate pH in the isolated, perfused guinea pig heart. Circ Res. 1978;42:225–229. 23224 10.1161/01.res.42.2.225

[32] Ishizaka H , Kuo L . Acidosis‐induced coronary arteriolar dilation is mediated by ATP‐sensitive potassium channels in vascular smooth muscle. Circ Res. 1996;78:50–57. 8603505 10.1161/01.res.78.1.50

[33] de Brito‐Ashurst I , Varagunam M , Raftery MJ , Yaqoob MM . Bicarbonate supplementation slows progression of CKD and improves nutritional status. J Am Soc Nephrol. 2009;20:2075–2084. 19608703 10.1681/ASN.2008111205 PMC2736774

[34] Merten GJ , Burgess WP , Gray LV , Holleman JH , Roush TS , Kowalchuk GJ , Bersin RM , Van Moore A , Simonton CA III , Rittase RA , Norton HJ , Kennedy TP . Prevention of contrast‐induced nephropathy with sodium bicarbonate: a randomized controlled trial. JAMA. 2004;291:2328–2334. 15150204 10.1001/jama.291.19.2328

[35] Recio‐Mayoral A , Chaparro M , Prado B , Cozar R , Mendez I , Banerjee D , Kaski JC , Cubero J , Cruz JM . The reno‐protective effect of hydration with sodium bicarbonate plus N‐acetylcysteine in patients undergoing emergency percutaneous coronary intervention: the RENO Study. J Am Coll Cardiol. 2007;49:1283–1288. 17394959 10.1016/j.jacc.2006.11.034

[36] Brar SS , Shen AY , Jorgensen MB , Kotlewski A , Aharonian VJ , Desai N , Ree M , Shah AI , Burchette RJ . Sodium bicarbonate vs sodium chloride for the prevention of contrast medium‐induced nephropathy in patients undergoing coronary angiography: a randomized trial. JAMA. 2008;300:1038–1046. 18768415 10.1001/jama.300.9.1038

[37] Maioli M , Toso A , Leoncini M , Gallopin M , Tedeschi D , Micheletti C , Bellandi F . Sodium bicarbonate versus saline for the prevention of contrast‐induced nephropathy in patients with renal dysfunction undergoing coronary angiography or intervention. J Am Coll Cardiol. 2008;52:599–604. 18702961 10.1016/j.jacc.2008.05.026

[38] Thayssen P , Lassen JF , Jensen SE , Hansen KN , Hansen HS , Christiansen EH , Junker A , Ravkilde J , Thuesen L , Veien KT , Jensen LO . Prevention of contrast‐induced nephropathy with N‐acetylcysteine or sodium bicarbonate in patients with ST‐segment‐myocardial infarction: a prospective, randomized, open‐labeled trial. Circ Cardiovasc Interv. 2014;7:216–224. 24714489 10.1161/CIRCINTERVENTIONS.113.000653

[39] Klima T , Christ A , Marana I , Kalbermatter S , Uthoff H , Burri E , Hartwiger S , Schindler C , Breidthardt T , Marenzi G , Mueller C . Sodium chloride vs. sodium bicarbonate for the prevention of contrast medium‐induced nephropathy: a randomized controlled trial. Eur Heart J. 2012;33:2071–2079. 22267245 10.1093/eurheartj/ehr501

